# The devil is in the details: Genomics of transmissible cancers in Tasmanian devils

**DOI:** 10.1371/journal.ppat.1007098

**Published:** 2018-08-02

**Authors:** Andrew Storfer, Paul A. Hohenlohe, Mark J. Margres, Austin Patton, Alexandra K. Fraik, Matthew Lawrance, Lauren E. Ricci, Amanda R. Stahlke, Hamish I. McCallum, Menna E. Jones

**Affiliations:** 1 School of Biological Sciences, Washington State University, Pullman, Washington, United States of America; 2 Department of Biological Sciences, University of Idaho, Moscow, Idaho, United States of America; 3 Institute for Bioinformatics and Evolutionary Studies, University of Idaho, Moscow, Idaho, United States of America; 4 School of the Environment, Griffith University, Nathan, Australia; 5 School of Biological Sciences, University of Tasmania, Hobart, Australia; University of Michigan Medical School, UNITED STATES

Cancer poses one of the greatest human health threats of our time. Fortunately, aside from a few rare cases of cancer transmission in immune-suppressed organ transplant recipients [1] or a small number of transmission events from mother to fetus [2], cancers are not spread from human to human. However, transmissible cancers have been detected in vertebrate and invertebrate animals, sometimes with devastating effects [3]. Four examples of transmissible cancers are now known: 1) canine transmissible venereal tumor (CTVT) in dogs [4], 2) a tumor in a laboratory population of Syrian hamsters that is no longer cultured [3], 3) infectious neoplasias in at least four species of bivalve mollusks [5,6], and 4) two independently derived transmissible cancers (devil facial tumor disease [DFTD]) in Tasmanian devils [7–10] (Fig 1A and 1B). The etiologic agents of CTVT [4], the bivalve cancers [5], and DFTD [7] are the transplants (allografts) of the neoplastic cells themselves, but the etiologic agent is unknown for the hamster tumor.

**Fig 1:**
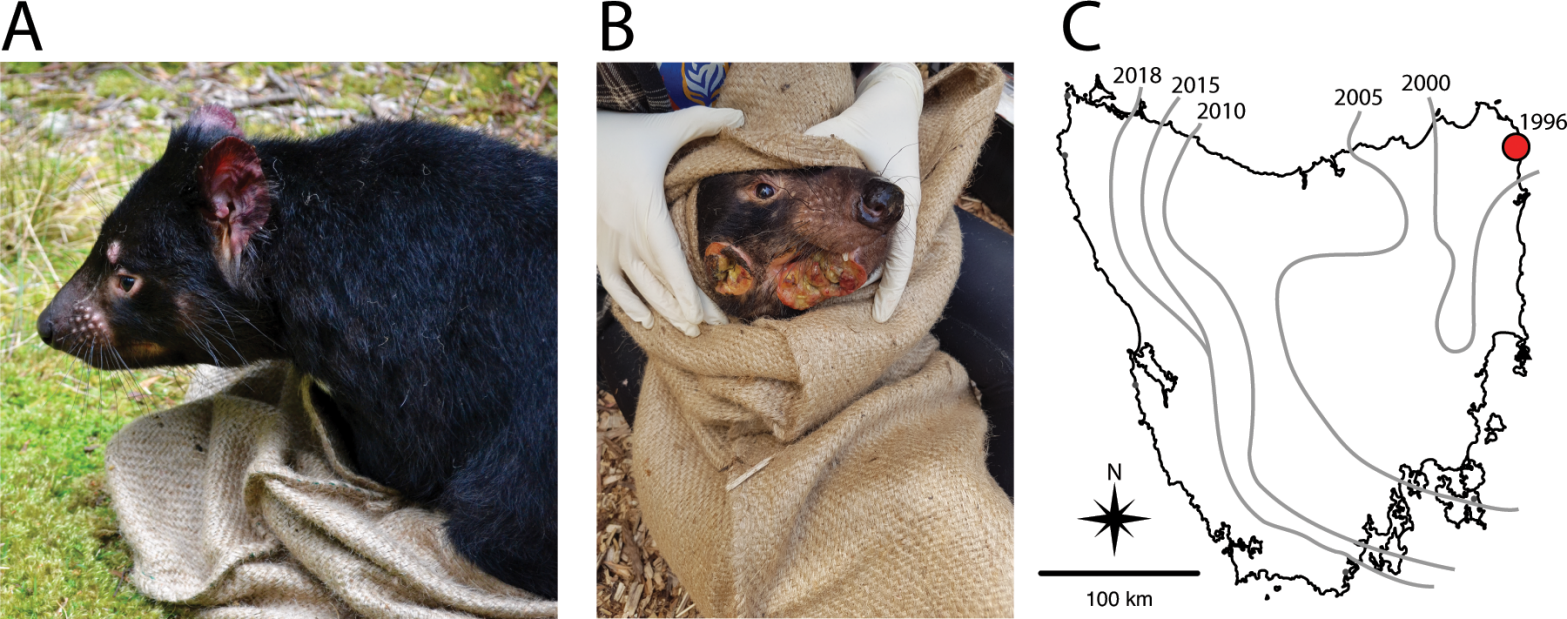
A. Healthy adult Tasmanian devil. B. Adult Tasmanian devil infected with devil facial tumor disease. C. Map of Tasmania showing initial site of disease emergence (red dot). Curved lines show the disease front demarcated by the year devil facial tumor disease was detected in each area. *Photo Credit*: *Alexandra Fraik*.

The effects of these transmissible cancers on their respective host populations vary. CTVT is spread in dogs through sexual contact and is at least 11,000 years old, placing the timing of its origin close to that of the domestication of dogs [11]. Although genomic analyses of the tumor suggest evasion of multiple components of the dog immune system, dogs most commonly survive and often show evidence of spontaneous tumor regression within a year of initial diagnosis [11,12]. For the infectious bivalve neoplasias, which have existed for at least 40 years, population effects vary from enzootic infections with no noticeable effects on population sizes to evidence of a catastrophic population decline [6,13]. In Tasmanian devils (Fig 1A), the first infectious tumor discovered (DFT1; Fig 1B) has spread across approximately 95% of the geographic range of Tasmanian devils since 1996 (Fig 1C). DFTD is almost always fatal (Fig 1B), with >90% declines in infected localities and an overall species-wide decline exceeding 80% [14–16]. Transmission dynamics appear consistent with frequency dependence, with DFTD spread by biting during social interactions [15], resulting in predictions of extinction from standard epidemiological models [14]. Despite these predictions, long-infected devil populations persist at reduced densities, suggesting that individual-level variability in fecundity and tumor growth rate in infected individuals are key for understanding epidemiological dynamics [17]. Additionally, the origin of the second, independent lineage of DFTD (i.e., DFT2) [9,10] within 20 years of the discovery of DFT1 suggests that transmissible cancers may be a recurring part of the Tasmanian devils' evolutionary history, without causing extinction [17].

## The origin of Tasmanian devil facial tumor disease

Based on a transcriptomic analysis of DFT1, the progenitor tumor likely originated from a mutated Schwann cell (a type of peripheral nerve cell) in a female Tasmanian devil [8]. DFT2 is also likely to be of neuroectodermal origin, but DFT2 does not express periaxin (PRX), a Schwann cell marker present in DFT1 [10]. Both DFT1 and DFT2 likely evolved from Tasmanian devils located in eastern Tasmania, with their genetic assignments consistent with their geographic origins (in the NE and SE, respectively) [10]. The gross morphology and histology of DFT2 are different from DFT1 [9,10]. For example, DFT1 is generally composed of pleomorphic round cells in bundles, whereas DFT2 is typically characterized by pleomorphic sheets of cells [9]. Moreover, DFT2 karyotypes have a Y chromosome, indicating that this tumor arose from a male devil and thus independently from DFT1 [9]. Although DFT1 and DFT2 originated in the last 21 years, no evidence has been found for viral origin, and results are inconsistent with tumor evolution coming from exposure to anthropogenic stressors, such as increased UV light [10].

**Fig 2:**
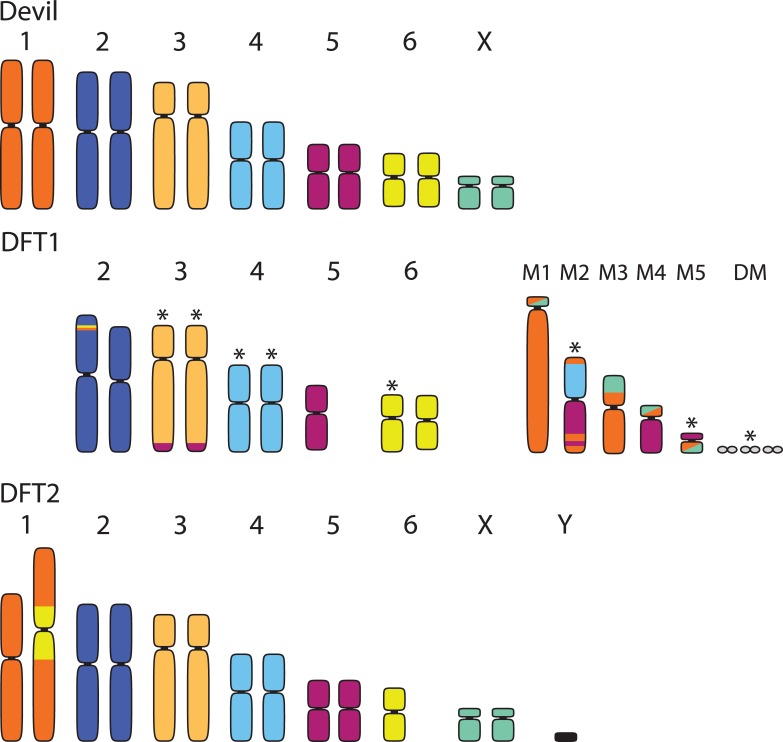
Karyotypes of a normal female Tasmanian devil (top) with a representative karyotype of DFT1 (middle) and DFT2 (bottom). Karyotypes of the Tasmanian devil and DFT1 follow [19]; this arrangement differs from [10] in respect to Chromosomes 1 and 2, in which the positions are reversed. Colors in rearranged/fragmented chromosomes indicate the chromosome from which the material originated in the normal devil karyotype. M1–M5 indicate marker chromosomes resulting from fragmentation of Chromosome 1 and fusion with Chromosomes 5 and 1 in DFT1. Asterisks indicate those chromosomes/markers that have been observed to vary among karyotyped strains of DFT1. DFT2 originated from a male devil and thus possesses a Y chromosome. While one copy of Chromosome 6 has been inserted into the pericentric region of Chromosome 1, the limited available karyotypes of DFT2 thus far suggest it is otherwise relatively undifferentiated from the normal devil karyotype. DFT, devil facial tumor.

Although the specific mechanism of how DFT1 or DFT2 became transmissible is still unknown, several lines of evidence suggest that chromosomal rearrangements, common to cancers, were critical [8,18]. Devils normally have six pairs of autosomes and one pair of sex chromosomes [7,19,20], but cytogenetic analyses show large-scale rearrangements in both DFT1 and DFT2 (Fig 2). To date, few isolates of DFT2 have been karyotyped, but DFT1 has abnormalities associated with Chromosomes 1, 3, 4, 5, 6, and the X chromosome [19–21]. DFT1 is characterized by a series of marker chromosomes, including double minutes, that vary in size and number among different cytogenetic strains [10,21]. Notably, both tumors show rearrangement of Chromosome 1 (referred to as Chromosome 2 in [10]; see Fig 2) [20,21]. Some karyotypes of DFT1 show complete fragmentation of Chromosome 1, as well as fusion with parts of the X chromosome and Chromosome 5, whereas DFT2 has an insertion of one copy of Chromosome 6 in the pericentric region in Chromosome 1 (Fig 2). Comparisons with wallaby and opossum genomes suggest that Chromosome 1 has been prone to rearrangement among marsupials [20]; Chromosome 1 rearrangements are stable in DFT1, indicating their possible importance for tumor transmissibility and fitness [19,20]. Common to cancers, DFT1 and DFT2 may have arisen from a critical shortening of telomeres on different chromosomes that led to these abnormal karyotypes [see 10,19,20]. Telomeres are DNA–protein complexes that protect against chromosomal degradation during cell replication [22]. When telomeres become critically short, the p53 pathway typically induces apoptosis (terminal cell cycle arrest), but evasion of this pathway is often associated with cancer progression [23].

At least 64 chromosomal rearrangements map to genic regions in DFT1, including a gene encoding a histone methyltransferase (*EZH2*) that is dysregulated in many cancers [10]. Additional translocations include a homolog of a tumor suppressor gene (*NF2*) and a deletion of a tumor suppressor copy (*LZTR1*). Moreover, there is evidence of copy number increases of two genes (*ERBB3* and *GALNT2*) that are overexpressed in many cancers, including schwannomas [18,20]. While early mutations in DFT1 and DFT2 have no overlap, both tumors notably had hemizygous mutations that led to deletion of second copies of genes in the Hippo pathway (*WWC* in DFT1 and *MPDZ* in DFT2, respectively), implicated in several human cancer histotypes, particularly Schwann cell cancers [10]. Although both tumors are largely diploid, DFT2 appears to have a simpler structure and fewer rearrangements (23 versus 64) as compared to DFT1 [10].

## Tasmanian devil facial tumor disease: Genomics of susceptibility

For an allograft to avoid rejection from a new host, it must circumvent recognition by major histocompatibility complex (MHC) genes [24]. MHC Class I is generally responsible for tumor recognition via identification of cell surface proteins expressed as “nonself” on cancer cells [24]; transmissible cancer cells are indeed nonself, having originated in a different individual [3,6]. Ubiquitous susceptibility of Tasmanian devils to DFTD has been hypothesized to result from low devil genetic variability overall [25], likely due to at least two historical genetic bottlenecks [25,26]. Compared to other mammals, devils have particularly low genetic variability in the MHC Class I peptide-binding region implicated in tumor recognition [27]. However, MHC diversity is not linked to variation in disease susceptibility among individuals [28], and devils reject allografts in challenge experiments [29]. Instead, DFT1 appears to down-regulate its own MHC expression, as well as MHC expression in the devil [30]. Epigenetic down-regulation of MHC expression is common in human cancers [31], as well as being a salient feature in CTVT [12,18]. In addition to MHC evasion, there are at least several other mechanisms that underlie widespread transmissibility of DFTD yet to be discovered. Moreover, there is documented variation in tumor susceptibility among devils, including rare documented cases of tumor regression and immune response, which are discussed below.

## Tasmanian devil facial tumor disease: Evolution of devils

Despite widespread declines of Tasmanian devils and predictions of localized devil extinctions, continued devil survival may result from evolution of DFTD resistance, which is supported by multiple lines of genetic evidence. First, a genome scan showed large and concordant allele frequency changes and increases in linkage disequilibrium across three populations pre- and post-disease [32]. Strong support for rapid evolution (in as few as four generations) was discovered in two small genomic regions containing seven candidate genes mapped to the devil reference genome; five of these genes were associated with immune- and cancer-related functions, including cell adhesion and p53 pathways [32]. Second, a genome-wide association study showed strong evidence that a few large-effect single nucleotide polymorphisms (SNPs or single base pair changes in DNA) explain a significant proportion of observed phenotypic variation in survival following infection in females [33]. Genes of particular interest in close proximity with these SNPs also include cell adhesion, tumor suppression, and p53 pathway genes [33]. Taken together, these two studies suggest evolution resulting from a soft selective sweep, whereby selection acted on standing genetic variation in a few, large-effect loci, as opposed to on new mutations [33]. A third study showed that DFTD was capable of swamping local adaptation to weaker abiotic forces, such as altitude [34]. That is, selection by the biotic factor of disease tended to overwhelm selection by abiotic factors in the predisease environment [34].

Recent field studies also suggest evolution of DFTD resistance. For the first time, spontaneous regression of tumors, a phenomenon rarely seen in human cancers without treatment, has been documented in devils [35]. A comparative genomic study of devils with tumor regression versus those that succumbed to the disease shows evidence that two devil candidate genes (*TLL1* and *NBAS1*) are involved in the regression process [35,36]. The two genes, plus a third (*PAX3*, which is not significant after genome-wide correction), are involved in stimulating angiogenesis in both normal growth and cancer metastases, perhaps increasing tumor vascularization to enable lymphocyte penetration. Indeed, Pye and colleagues [36], in the first study that demonstrates evidence of adaptive immune response to DFTD, show that one devil had prominent lymphocyte infiltration in its tumor. The same study shows presence of serum antibodies against DFTD cells in six of 52 devils tested, and that four of these six devils had histories of tumor regression [36].

## Tasmanian devil facial tumor disease: Evolution of the tumors

Cytogenetic analyses currently recognize four karyotypes of DFT1 [21] and show that genomic rearrangements are limited to particular cancer regions, suggesting at least some genomic stability [19,20]. When compared to the Tasmanian devil reference genome, two strains of DFT1 collected from SE and north central Tasmania, respectively, accumulated between 15,000 and 17,000 single-nucleotide substitutions between them [18]. Assuming they share a common ancestor that emerged approximately 20 years ago, this mutation rate is higher than most human cancers (approximately 5,000) but lower than lung cancer or melanomas [18]. Evidence of within-host tumor variation is limited; of 20 devils with multiple tumors, only six individuals had tumors that were genetically distinguishable. A comparison of the mitochondrial genomes of 104 DFT1 tumors from 69 devils across Tasmania showed limited among-host genetic variability as well, with 21 somatic variants detected [18].

Overall, there appears to be variation in fitness across different DFT1 lineages. Evidence for this comes from displacement of one tumor lineage by another in at least one area of Tasmania [16] but coexistence of multiple lineages in other areas [18]. Currently, DFT2 is still limited in its geographic distribution, and it is not yet known how fast the lineage is evolving, nor how its fitness or effects on devil fitness compare with DFT1. However, early evidence suggests that DFT2 primarily infects males (9/11 documented cases), possibly indicating relative differences in susceptibility between males and females [10].

## Conclusion

Transmissible cancers are indeed a frightening phenomenon, and the recent appearance of a malignancy of tapeworm origin in an HIV-infected individual [37] shows that tumors derived from nonhost DNA can emerge spontaneously in immune-suppressed humans. As a species, the Tasmanian devil has perhaps suffered most extensively, with massive population declines resulting from the emergence and spread DFTD. Fortunately, a combination of genomic and immunological studies provide compelling evidence of devil evolutionary responses that appear to be related to DFTD resistance or tolerance. Further, an in vitro drug screen showed promise for possible oral treatment therapies; both DFT1 and DFT2 are highly sensitive to several clinical compounds, and DFTs apparently show low tolerance to DNA damage [10]. The development of numerous genomic data sets to study human cancers, DFTD, CTVT, and soon likely the bivalve neoplasias provides extensive resources for the study of cancer transmissibility. Further research that uses comparative genomics and transcriptomics, such as the recent comparison of DFT1 and DFT2 [10], will likely be fruitful for understanding the origin and evolution of cancer transmissibility in general.
